# Interruption of CXCL13-CXCR5 Axis Increases Upper Genital Tract Pathology and Activation of NKT Cells following Chlamydial Genital Infection

**DOI:** 10.1371/journal.pone.0047487

**Published:** 2012-11-26

**Authors:** Janina Jiang, Ouafae Karimi, Sander Ouburg, Cheryl I. Champion, Archana Khurana, Guangchao Liu, Amanda Freed, Jolein Pleijster, Nora Rozengurt, Jolande A. Land, Helja-Marja Surcel, Aila' Tiitinen, Jorma Paavonen, Mitchell Kronenberg, Servaas A. Morré, Kathleen A. Kelly

**Affiliations:** 1 Department of Pathology and Laboratory Medicine, David Geffen School of Medicine, University of California Los Angeles, Los Angeles, California, United States of America; 2 California NanoSystems, University of California Los Angeles, Los Angeles, California, United States of America; 3 CURE DDRC Morphology and Images Core, University of California Los Angeles, Los Angeles, California, United States of America; 4 Laboratory of Immunogenetics, Department of Medical Microbiology and Infection Control, VU University Medical Center, Amsterdam, The Netherlands; 5 Institute of Public Health Genomics, Department of Genetics and Cell Biology, Research Institutes, School of Public Health and Primary Care (CAPHRI) and Growth and Development (GROW), Faculty of Health, Medicine & Life Sciences, University of Maastricht, Maastricht, The Netherlands; 6 La Jolla Institute for Allergy and Immunology, La Jolla, California, United States of America; 7 Department of Obstetrics and Gynaecology, University Medical Center Groningen, Groningen, The Netherlands; 8 National Institute for Health and Welfare, Kastelli Research Centre, Oulu, Finland; 9 Department of Obstetrics and Gynecology, University Hospital, University of Helsinki, Helsinki, Finland; University of California Merced, United States of America

## Abstract

**Background:**

Regulation of immune responses is critical for controlling inflammation and disruption of this process can lead to tissue damage. We reported that CXCL13 was induced in fallopian tube tissue following *C. trachomatis* infection. Here, we examined the influence of the CXCL13-CXCR5 axis in chlamydial genital infection.

**Methodology and Principal Findings:**

Disruption of the CXCL13-CXCR5 axis by injecting anti-CXCL13 Ab to BALB/c mice or using *Cxcr5−/*− mice increased chronic inflammation in the upper genital tract (UGT; uterine horns and oviducts) after *Chlamydia muridarum* genital infection (GT). Further studies in *Cxcr5−/−* mice showed an elevation in bacterial burden in the GT and increased numbers of neutrophils, activated DCs and activated NKT cells early after infection. After resolution, we noted increased fibrosis and the accumulation of a variety of T cells subsets (CD4-IFNγ, CD4-IL-17, CD4-IL-10 & CD8-TNFα) in the oviducts. NKT cell depletion *in vitro* reduced IL-17α and various cytokines and chemokines, suggesting that activated NKT cells modulate neutrophils and DCs through cytokine/chemokine secretion. Further, chlamydial glycolipids directly activated two distinct types of NKT cell hybridomas in a cell-free CD1d presentation assay and genital infection of *Cd1d−/−* mice showed reduced oviduct inflammation compared to WT mice. CXCR5 involvement in pathology was also noted using single-nucleotide polymorphism analysis in *C. trachomatis* infected women attending a sub-fertility clinic. Women who developed tubal pathology after a *C. trachomatis* infection had a decrease in the frequency of *CXCR5* SNP +10950 T>C (rs3922).

**Conclusions/Significance:**

These experiments indicate that disruption of the CXCL13-CXCR5 axis permits increased activation of NKT cells by type I and type II glycolipids of *Chlamydia muridarum* and results in UGT pathology potentially through increased numbers of neutrophils and T cell subsets associated with UGT pathology. In addition, CXCR5 appears to contribute to inter-individual differences in human tubal pathology following *C. trachomatis* infection.

## Introduction

*Chlamydia trachomatis,* an obligate intracellular bacterium, causes the most cases of bacterial sexually transmitted infections (STIs) in the US resulting in about three million new cases annually [1]–[3]. Genital infection can lead to immune-mediated damage of the female reproductive organs and serious reproductive disability, including pelvic inflammatory disease (PID) that can result in chronic pelvic pain, ectopic pregnancy and infertility. Approximately 8% of females annually develop PID and this risk increases by 40–70% following re-infection [3], [4]. Although female infection is easily detected and treated with antibiotics, treated individuals can acquire another infection in six months implicating repeated inflammatory insults as a cause of PID and infertility [5]. However, the mechanism(s) which causes PID and infertility following chlamydial genital infection is not known.

The mouse model of *C. trachomatis* genital infection (*C. muridarum*) is used to reveal the underlying mechanism(s) for developing immune-mediated damage of the female reproductive organs following STIs. It is known that *C. muridarum* bacteria cause genital tract (GT) infections which trigger development of protective immune responses but infection also results in GT inflammation and is associated with neutrophils and CD8 cells that produce TNFα [6]–[8]. Immune-mediated damage can be quantitated in the mouse, is a measure of infertility and is termed upper genital tract (UGT) pathology [9]. The majority of genital infections are resolved by development of an anti-chlamydial Th1 response [10], [11].

NKT cells are innate-like T cells that rapidly respond to infection and regulate microbial immunity including *C. muridarum* lung and GT infection [12]–[15]. NKT cells require TCR ligation for activation to secrete an array of cytokines and chemokines [16], [17]. In addition, they also modulate immune outcomes by interacting with dendritic cells (DC), NK cells, T, B cells and plasmacytoid DC by cell-cell contact [12]. NKT cells are activated with CD1d-restricted glycolipid antigens and are classified into two subsets [16], [18]. Type I (classical or invariant, iNKT) NKT cells express an invariant TCR, Vα14-Jα18 in the mouse and the homolog, Vα24-Jα18, in humans [19]. The antigen receptors expressed by iNKT cells in mice and humans recognize exogenous glycolipids expressed by microbes that contain a common glycolipid structure, including the GLXA glycolipid of *C. muridarum* [13]. Type II NKT cells are less studied than iNKT cells, have a more diverse in TCR repertoire and do not recognize the prototypical iNKT cell activating antigen, α-galactosylceramide (α-GalCer) [19].

Chemokines have an established role in homing and directional migration but also have additional roles in the immune system including; development of lymphoid organs, cognate interaction, cell signaling, differentiation, cell survival and serve as growth factors. Likewise, the chemokine CXCL13 has additional roles beyond migration and disruption of the CXCL13-CXCR5 axis occurs in many diseases as well as chronic infections [20]–[23]. We recently reported that *C. trachomatis* induces expression of CXCL13, the ligand for CXCR5, in human fallopian tube tissue following infection [24]. Surprisingly, the mRNA for this chemokine was induced at higher levels (30-fold over mock infected controls) in comparison to more than 90 other cytokines and chemokines analyzed including IFNγ. In this report, we examined the influence of the CXCL13-CXCR5 axis in chlamydial genital infection.

## Materials and Methods

### Ethics Statement

All experimental animal procedures were approved by the UCLA Office of Animal Research Oversight; Chancellor's Animal Research Committee which adheres to the national guidelines with the Public Health Service Policy on Human Care and Use of Animals (PHS Policy) and USDA Animal Welfare Regulation (AWRs) with assurance number A3196. All procedures were designed to provide for maximum comfort/minimal stress to the animals and cannot be further refined to minimize pain/distress since there are no less painful/distressful options available. The procedures are presently refined to provide the best possible scientific methodologies available. The animals are monitored for signs of agitation (licking, biting or guarding the vaginal region), failure to groom, loss of appetite, or marked weight loss (>10%). If noted, the Attending Veterinarian is contacted for his/her recommendation for treatment. Human subjects analyzed for CXCR5 SNP typing were consented by a written informed consent form and approved by review boards at Vrije Universiteit, Amsterdam, The Netherlands and University of Helsinki.

### Animals, Chlamydia, anti-CXCL13 treatment and Challenge of mice

BALB/c and C57BL/6 mice (Jackson Labs, Bar Harbor, Maine) or a breeding colony was established with *Cxcr5*−/− mice (8 generations in C57BL/6) obtained from Martin Lipp, Delbrück-Center for Molecular Medicine, Berlin, Germany. BALB/c mice were given two intraperitoneal injections of affinity purified goat IgG anti-CXCL13 or control goat IgG (R&D Systems, Minneapolis, Minn) on days −1 and 2 of infection [25]. *Chlamydia muridarum* was grown on confluent McCoy cell monolayers, purified on Renograffin gradients and stored at −80°C in SPG buffer (sucrose-phosphate-glutamine) as previously described [26]. Mice were hormonally synchronized by subcutaneous infection with 2.5 mg of medroxyprogesterone acetate (Depo Provera, Upjohn, Kalamazoo, MI) in 100 µL saline 7 days prior to a vaginal challenge with 1.5×10^5^ IFUs of *C. muridarum* under anesthetization. Mice were 5–6 weeks old at the time of infection. Infection was monitored by measuring infection forming units (IFU) from cervical–vaginal swabs (Dacroswab Type 1, Spectrum Labs, Rancho Dominguez, CA) as previously described [27].

### Histology, Hematoxylin & Eosin and Trichrome stain

GT's were removed at day 49 post-infection, fixed in 10% formalin overnight, and subsequently, 70% ethanol. Tissues were embedded en bloc in paraffin, sectioned (5 µm), and stained with hematoxylin and eosin. A second set was stained with hematoxylin and eosin followed by Gormori trichrome [28]. Two hematoxylin and eosin (H&E) sections per mouse were masked and scored for chronic inflammatory cells or mononuclear cells [29]: 0 = normal, 1 = rare foci, 2 = scattered (1 to 4) aggregates, 3 = numerous aggregates (>4), 4 = severe diffuse infiltration. Two slides were scored and averaged per oviduct. Trichrome stained slides were used to access fibrosis and were masked and counted using the following scoring scheme: 1+ = bright blue staining collagen fibers surrounding <33% of oviducts; 2+ = bright blue staining collagen fibers surrounding 34–66% of oviducts; 3+ = bright blue staining collagen fibers surrounding >66% of oviducts. Naïve mice score from 1–2+. Two slides were scored and averaged per oviduct.

### Lympholyte isolation and FACS identification

Spleen (Spl), iliac lymph nodes (ILN), GTs or oviducts were harvested from individual mice and single cell suspensions prepared by dissociating the lymphocytes. Lymphocyte isolation from GTs or oviducts was carried out as described [30]. Briefly, the entire GT or oviduct was removed and cut into 0.5 cm pieces that were then rinsed with Ca^2+^Mg^2+^-free Hanks' balanced salt solution (HBSS). The tissue was incubated in a mixture of 5 mM EDTA in HBSS at 37°C for two 15 min periods with gentle stirring. The tissue was then incubated with RPMI 1640 containing 2% bovine calf serum, antibiotics, 25 mM HEPES and 1.5 mg/mL collagenase (Sigma, USA) and incubated at 37°C with stirring for two periods of 1 hr. The isolated cells were pooled together and separated on a 40/75% discontinuous Percoll gradient (Pharmacia, Piscataway, N.J.) centrifuged at 2000 rpm at 22°C for 20 min. Mononuclear cell pellets were resuspended in RPMI 1640 at 4°C until used. For intracellular cytokine staining, lymphocytes isolated from different organs were incubated in RPMI 1640 in the presence of PMA and ionomycin. Brefeldin A (Sigma) was added 4 hr before the end of culture. For surface staining, cells were directly stained with various FACS antibodies after isolation and without *in vitro* culture. The cells were then stained with fluorochrome-labeled antibodies against CD3 (clone 145-2C11), CD4 (clone GK1.5), CD8 (clone 53-6.7), Gr-1 (clone RB6-8C5), NK1.1, (clone PK136), CD11c (clone N418), CD19 (clone 6D5), CD11b (clone…M1/70), CD40 (clone 3/23), CD69 (clone H1.2F3) and CXCR5 (clone 2G8) (Biolegend) and α-GalCer-CD1d-tetramer for 20 min on ice [31]. After being washed, the cells were incubated with Cytofix/Cytoperm (BD Biosciences) for 1 hr and the stained with fluorochrome-conjugated anti-IFN-γ (clone XMG1.2), IL-10 (clone ES5-16E3), TNFα (clone MP6-XT22) or IL-17 (clone TC11-18H10.1) antibody (Biolegend) for 20 min on ice, washed again, resuspended in Cell Fix solution, and analyzed on SORP BD LSR II (Beckman Dickinson, Franklin Lakes, NJ).

### NKT cell depletion, CD1d blocking and in vitro cell culture

Spleens were treated with iNKT depletion, CD1d blocking, iNKT depletion+CD1d blocking, or with α-GalCer (250 ng/mL) and untreated cells served as control. To deplete iNKT cells, splenocytes were stained with PE-α-GalCer-CD1d-tetramer on ice for 30 min and the stained iNKT cells were depleted by using EasySep Mouse EP Positive Selection Kit (Stemcell Technologies, Canada) according to manufacturer's protocol. The depletion efficiency was greater than 90% as tested by FACS analysis. To deplete CD1d presenting cells, the cells were treated with anti-CD1d monoclonal antibody (clone 1B1) (10 µg/mL) or isotype control for 30 min prior to culture. Cells were then cultured in RPMI 1640 in presence of *C. muridarum* EB for 3 days and supernatants were stored at −80°C.

### Cytokine measurement and multi-analyte ELISArray

IFNγ was measured by ELISA (R&D Systems) in supernatants from cell cultures following storage −80°C. IL-1β, IL-12, IL-6, CCL3, CCL4, CCL2, IL-17α, CXCL12, CCL22, CCL5 and TNFα were analyzed by multi-analyte ELISArray kit (SABiosciences, Frederick, MD) in supernatants from cell cultures following storage −80°C. The assays were carried out according to manufacturer's protocol and calibrated standards were provided by the manufacturer.

### In vitro CD1d presentation assay

We used the method of Kinjo et. al. [32] and first coated a 96-well flat bottom plate with soluble mCD1d protein for 1 h at 37°C. After washing and blocking, various concentrations of sonicated *C. muridarum* was added to wells and incubated for 24 h at 37°C. α-GalCer and vehicle served as positive and negative controls, respectively. The wells were washed and 5×10^4^ murine invariant NKT hybridoma cells: 1.2, 1.4, or variant NKT hybridoma 19 were added and cultured overnight (16–20 hr). IL-2 released in the supernatant upon activation was measured by ELISA (BD Pharmingen).

### Patients

#### Dutch STD cohort

Women of Dutch Caucasian origin (n = 543) visiting the STD outpatient clinic in Amsterdam, the Netherlands during the period of July 2001–December 2004. Participants were asked to sign an informed consent and fill out a questionnaire, regarding their complaints at that moment, varying from increased discharge, having bloody discharge during and/or after coitus, recent abdominal pain (not gastrointestinal or menses related) and/or dysuria. A cervical swab was taken for the detection of *C. trachomatis*-DNA by PCR [33]. Peripheral venous blood was collected for the analysis of IgG antibodies against *C. trachomatis* (Medac Diagnostika mbH, Hamburg, Germany). A titre of ≥1∶50 was considered positive. Those who had no *Chlamydia* infection, based on negative *C. trachomatis*-DNA and without *C. trachomatis* serology, served as controls. Infections with *Candida albicans, Neisseria gonorrhoea, Trichomonas vaginalis* and *Herpes simplex virus 1 or 2* were also documented. Microorganism detection was done according to methods described by Ouburg *et al.* [34].

#### Dutch Subfertility cohort

The study cohort included 259 Dutch Caucasian women who presented with subfertility at the Research Institute Growth and Development and the Department of Obstetrics and Gynaecology, University of Maastricht, The Netherlands, as part of a larger study. This subfertility group has been described elsewhere [34], [35]. For this study, women with or without clinically defined tubal pathology (n = 56) were selected to have the clearest clinical definition. Tubal pathology was defined as extensive periadnexal adhesions and/or distal occlusions of one or both tubes. Chlamydial antibodies were assessed by indirect microimmunofluorescence (MIF) test for anti-*C. trachomatis* IgG-antibodies. A positive CT IgG MIF test was defined as a titer ≥1∶32.

#### Finland study population

The study population consisted of 114 infertile women who had attended the *In Vitro* Fertilization Unit, Department of Obstetrics and Gynaecology, Helsinki University Hospital, Helsinki, Finland in 1990–2005. Controls were selected from a group of 176 female blood donors. The selected cases consisted of 24 *C. trachomatis*-positive women with laparoscopically verified tubal factor infertility (TFI) for the best clinical definition. The other women, the control group - consisted of female blood donors whose buffy coat specimens were provided by the Finnish Red Cross Blood Transfusion Service (Oulu, Finland). The specimens were transported to the laboratory at room temperature within 24 h of donation. The specific immune responses of *C. trachomatis* have been described elsewhere and *C. trachomatis* status was determined by *C. trachomatis* specific serology and cell stimulation as described previously [36].

#### Cxcr5 SNP detection

Genomic DNA was extracted from peripheral blood using the MagNaPure LC isolator as described [34]. A healthy Dutch Caucasian control group (n = 130) was included to assess the general frequency of the three studied SNPs *CXCR5* +3439 C>T (rs497916), +9086 T>C (rs12363277), +10950 T>C (rs3922) in the general Dutch Caucasian population. The *CXCR5* SNPs were determined using the standard TaqMan analysis. The three *CXCR5* SNPs tag 96.6% of the haplotypes, consisting of rs497916, rs566416, rs543524, rs12363277, rs613791, rs598207, rs3922, and rs676925. This haplotype spans 7 KB of the *cxcr5* gene.

#### Statistics

Bonferroni's modified *t*-test and analysis of variance (ANOVA) was performed using GraphPad Prizm version 5.04, GraphPad Software (San Diego, CA). Groups were considered statistically different at *p<0.05 and **p<0.01. Data are presented as mean ± SD or SEM as indicated. Differences in human *CXCR5* genotype and haplotype distributions were analyzed using χ^2^ and Fisher Exact test, where appropriate and p<0.05 was considered statistically significant. *CXCR5* haplotypes were inferred using PHASE v2.1.1 and SNPHAP.

## Results

### Disruption of the CXCL13-CXCR5 axis alters bacterial burden in *Cxcr5−/−* mice

**Figure 1:**
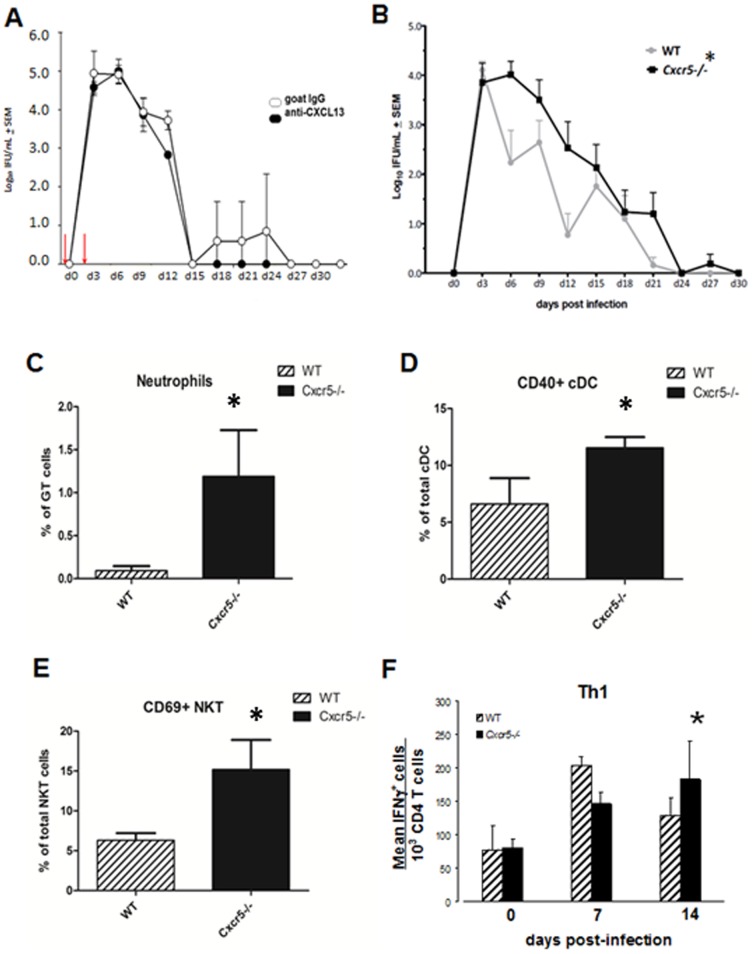
Disruption of the CXCL13-CXCR5 axis alters bacterial burden in *Cxcr5−/−* mice. Vaginal swabs were collected throughout the course of infection in BALB/c mice given i.p. injections (day −1 and 2, red arrow) of anti-CXCL13 Ab or irrelevant control Ab. Not significant by 2-way repeated measures ANOVA, n = 3/grp. (B) Vaginal swabs were collected throughout the course of infection in *Cxcr5−/−* and WT mice. *p<0.05 by 2-way repeated measures ANOVA, n = 11/grp. Experiments were and repeated 5 times. (C–E) Single cell suspensions of whole GT lymphocytes were stained for flow cytometric identification of neutrophils (Gr-1^+^ & CD11b^+^); cDC (CD11c^+^, CD11b^+^, CD3^−^, CD19^−^ CD40^+^) and activated NKT cells (α-GalCer tetramer, NK1.1^+^, CD3^+^; CD69^+^). Dotplots were gated on singlet lymphocytes and the above markers. Bar graphs show the percent of neutrophils, CD40^+^cDC and CD69^+^NKT over the indicted denominator ± SD in GT. *p<0.01 as determined by Bonferroni's t-test, n = 4 pools of two mice/group. (F) Mice were vaginally infected with *C. muridarum* and lymphocytes were isolated at various times following infection from the whole GT. Cells were stimulated *ex vivo* with PMA and ionomycin and characterized by flow cytometry. Dotplots were gated on CD3 and CD4. Bars show the mean frequency of Th1, IFNγ^+^ cells per group ± SD of 4 mice/group. *p<0.05 as determined by Bonferroni's modified t-test.

We evaluated the chlamydial burden in the GT from vaginal swabs by using two models for disrupting the CXCL13-CXCR5 axis. In the first model, we blocked ligation of CXCL13 with its receptor, CXCR5, by administering anti-CXCL13 as reported [25]. As shown in Figure 1A, mice were given 200 µg of polyclonal goat anti-CXCL13 and irrelevant polyclonal goat Ab the day prior to infection (d-1) and 2 days after infection (red arrows). This regimen did not produce any statistical difference in the course of infection between the groups although we noted that anti-CXCL13 treated mice cleared infection a few days earlier than controls. This procedure does not show depletion of CXCL13 in ELISA assays (data not shown) and there is no assay to detect functional blocking *in vivo* [25]. We also examined *Cxcr5−/−* mice and found that there was a slight delay in resolution of infection and a significant increase in bacterial burden during the infection course compared to WT controls (Fig. 1B). Consistent with the increased bacterial burden we also found increases in early immune responders such as neutrophils, activated CD40^+^ conventional DC (cDC) and activated CD69^+^ NKT cells in the GT (Fig. 1C–E) but no difference was seen in the number of plasmacytoid DC cells (data not shown) which also responds to *C. muridarum* genital infection [37]. *Cxcr5−/−* mice lack Peyer's patches, axillary, inguinal, parathymic, mediastinal and iliac lymph nodes but have facial, cervical, mesenteric lymph nodes and spleen [38], [39]. Possibly, the lack of iliac lymph nodes could delay the appearance of Th1 cells in the GT and also effect bacterial load. In support, we found that *C. muridarum* responsive Th1 cells peak earlier on day 7 in WT mice compared to day 14 of *Cxcr5/−* mice (Fig. 1F).

### Disruption of the CXCL13-CXCR5 axis increased UGT pathology

**Figure 2:**
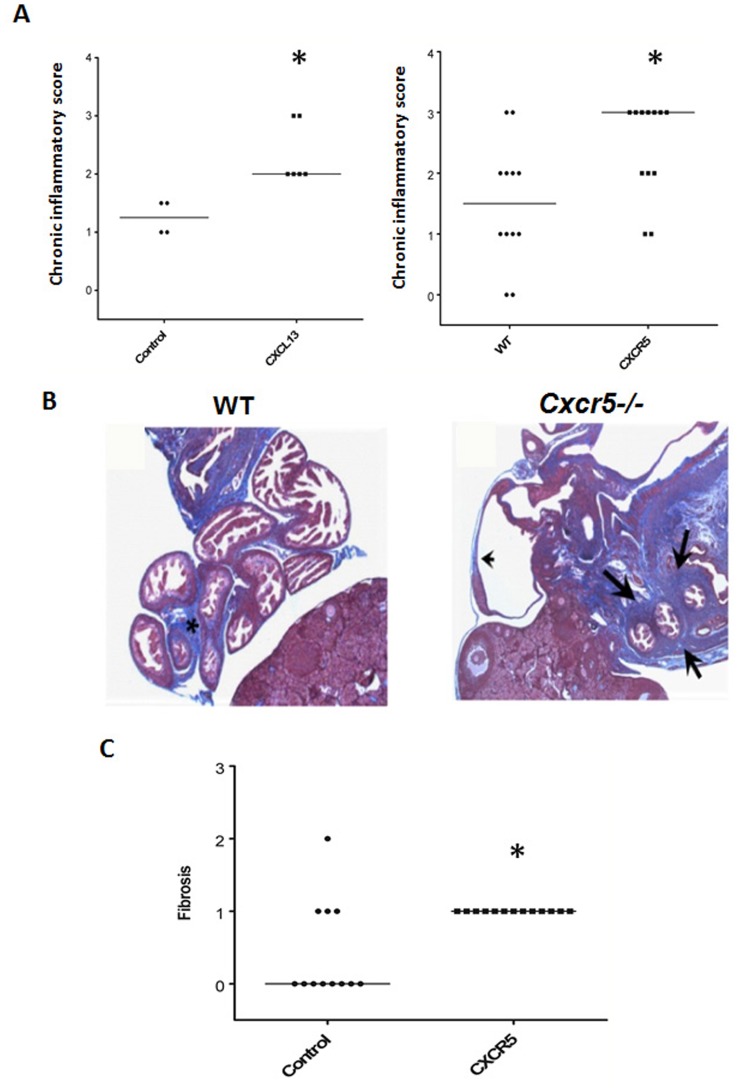
Disruption of the CXCL13-CXCR5 axis increased UGT pathology. GTs were harvested 49 days after infection and processed en bloc for paraffin sections. (A) Scatter plot and median of chronic inflammatory scores of BALB/c mice treated with anti-CXCL13 Ab versus an irrelevant control Ab or C56BL/6 WT mice versus *Cxcr5−/−* mice from hematoxylin and eosin stained slides. *p<0.05, Mann-Whitney, n = 3–6 mice or 12 oviducts/group. (B) Photomicrograph of trichrome stained sections from oviducts 49 days after infection of C57BL/6 (WT) or *Cxcr5*−/− mice. Asterisk (WT) and arrows (*Cxcr5−/−*) show fibrosis and arrowhead (*Cxcr5−/−*) depicts a dilated oviduct. Sections are at 20×. (C) Fibrosis scores of C56BL/6 WT mice and *Cxcr5−/−* mice from trichrome stained slides. *p<0.01, Mann-Whitney, n = 6 mice or 12 oviducts/group.

**Figure 3:**
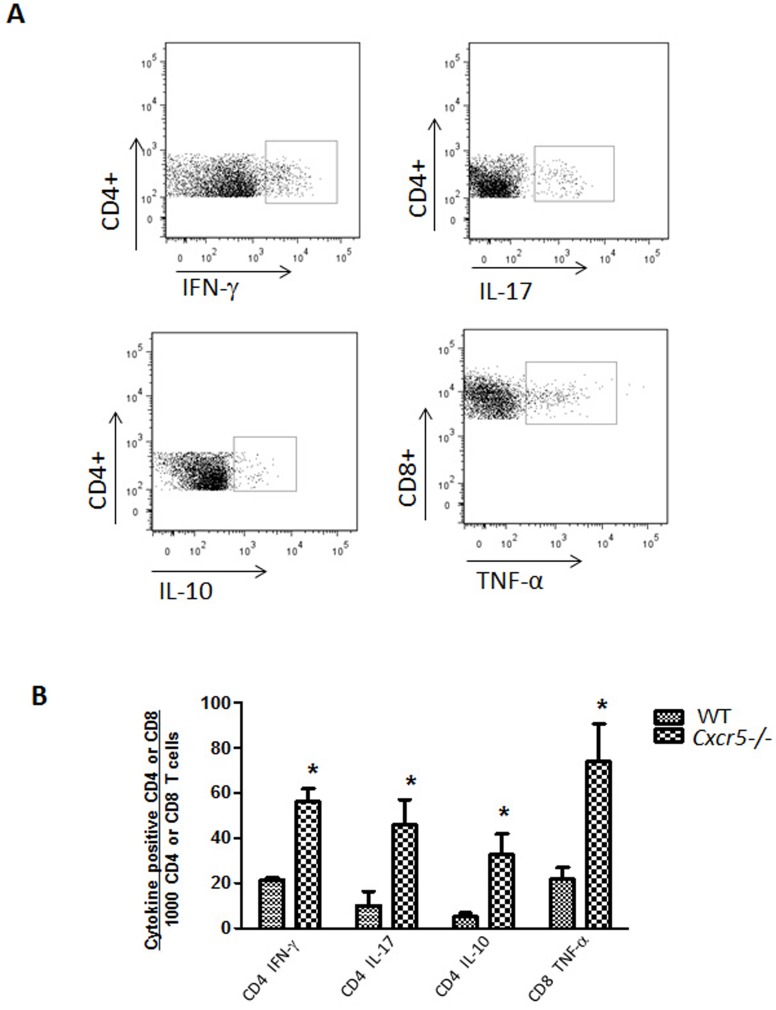
Lymphocytes accumulate in the oviducts of *Cxcr5*−/− mice following infection. GTs were harvested 49 days after vaginal infection with *C. muridarum* and used to isolate lymphocytes from the oviducts. (A) Representative dotplots of oviduct lymphocyte subsets stained against surface markers and intracellular cytokines. Cells were gated on CD3^+^CD4^+^ or CD3^+^CD8^+^ cells. (B) The number of lymphocyte subsets as determined in panel B, were compared between groups. Bars equal the mean ± SD. *p<0.01 (Bonferroni's modified t test). n = 3–4 oviduct pools/group.

We evaluated UGT (uterine horns+oviducts) pathology in mice treated with anti-CXCL13 Ab and *Cxcr5−/−* mice by examining inflammatory scores in the UGT from hematoxylin and eosin stained slides and fibrosis from trichrome stained slides of oviducts after infection. Mice treated with anti-CXCL13 Ab and *Cxcr5−/−* mice had increased chronic inflammatory or mononuclear cell scores when harvested 49 days after infection (Fig. 2A). We also measured the amount of fibrosis seen in mesenchymal tissue from WT and *Cxcr5−/−* mice. The mesenchymal tissue surrounding oviducts from WT mice developed varying degrees of fibrosis after infection (asterisk) which was increased in *Cxcr5−/−* mice (arrows) (Fig. 2B). Marked fibrosis was also apparent in all *Cxcr5−/−* mice following infection (Fig. 3C). Although we did not note occluding fibrosis which is associated with infertility, oviduct dilation was also noted in *Cxcr5−/−* (arrowhead)(Fig. 2B).

### T cell subsets accumulate in the oviducts of *Cxcr5*−/− mice following infection

**Figure 4:**
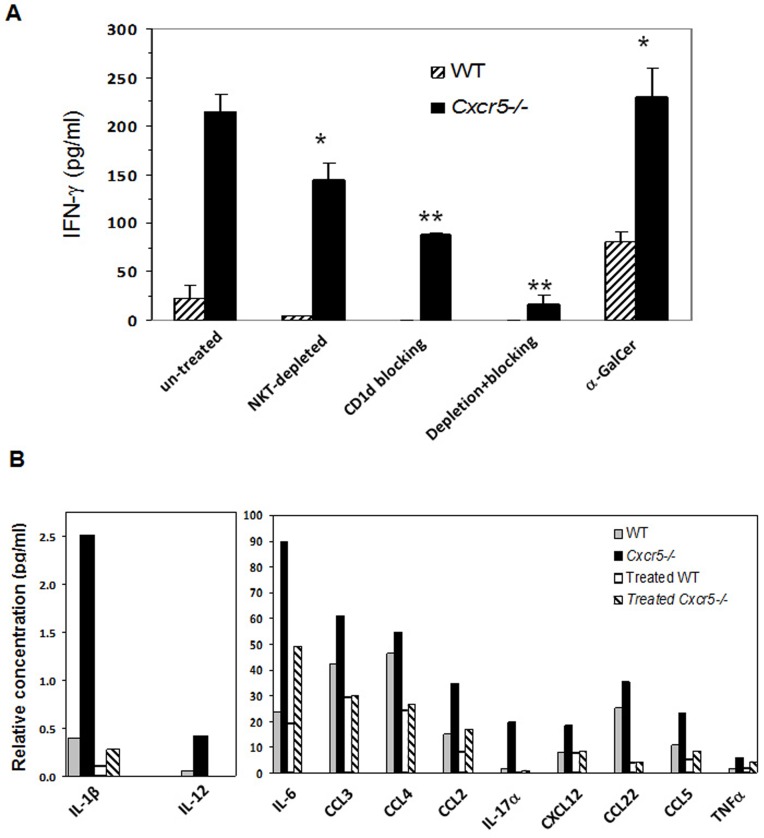
In vitro depletion of NKT cell function abrogates increased production of IFNγ and other cytokines and chemokines in *Cxcr5−/−*. Lymphocytes were isolated from spleens and treated with various conditions to deplete NKT cells to 95% purity. Cells were cultured with EB for 3 days. (A) The level of IFNγ in cell culture supernatants among groups with the indicated NKT cell depleting/blocking treatment. Each treated group was compared to its own untreated counterpart using Bonferroni's modified *t* test, *p<0.05, **p<0.01, n = 4 mice/group. (B) Multiple cytokines and chemokines levels were measured in supernatants from α-GalCer depleted plus anti-CD1d treated cell cultures (Depletion+blocking).

After chlamydial genital infection had resolved, we performed flow cytometry on CD4 and CD8 lymphocyte subsets from oviducts of *Cxcr5−/−* and WT mice to identify the type of accumulating mononuclear cells. Figure 3A shows representative dotplots of the subsets analyzed. We found a significant increase in all lymphocyte subsets analyzed in *Cxcr5−/−* mice which include; CD4-IFN-γ, CD4-IL-17, CD4-IL-10 and CD8-TNF-α (Fig. 4B). Larger increases were noted in CD4-IL-10 and CD8-TNFα subsets than the Th1 subset. Thus, mice lacking the CXCR5 chemokine receptor sustain a chronic infiltrate of a variety of T cell subsets. The CD4-IL-10 and CD8-TNFα subsets have been associated with reduced chlamydial clearance or oviduct pathology, respectively [8], [40]. These data suggest that increased numbers of activated cDC result in increased numbers of T cells in the GT.

### The lack of CXCR5 causes increased cytokine production by NKT cells

NKT cells respond as innate-like T cells within infected tissues by secreting an array of cytokines and chemokines upon TCR activation which results in the recruitment and/or activation of various immune cells including neutrophils and cDCs. This could possibly explain the increased neutrophil and activated cDC numbers seen in the GT of *Cxcr5−/−* 4 days after infection (Fig. 1C–E) [12]. We tested this by activating splenocytes *in vitro* with chlamydiae elementary bodies (EBs). Using IFN-γ production as a marker of early NKT cell activation, we found that splenic cultures from *Cxcr5−/−* mice produced approximately 10-fold greater amounts of IFN-γ compared to cultures from WT mice (Fig. 4A). We then used various means to deplete cultures of NKT cells in order to determine if NKT cells were the source of increased IFN-γ. Depletion of iNKT cells using the α-GalCer-CD1d tetramer, depleted greater than 90% of type I NKT cells and significantly diminished IFN-γ production. Stimulation with α-GalCer increased production of IFN-γ above that of WT cells and demonstrated that iNKT cells contribute in part, to the increased IFN-γ production. We examined IFNγ production from all NKT cells by blocking CD1d antigen presentation with anti-CD1d antibody. This further reduced IFN-γ levels. In addition, the combination of both treatments reduced IFN-γ production from *Cxcr5−/−* splenocytes to that of untreated WT control splenocyte cultures following incubation with *C. muridarum* EB which indicates that the majority of the IFNγ secreted by splenocytes in response to EB stimulation was secreted by iNKT and type II NKT cells (Fig. 4A).

We analyzed other cytokine and chemokines in the splenic cultures of *Cxcr5−/−* and WT cells stimulated with EB and found they produced a variety of inflammatory cytokines and chemokines that are found in the GT and that attract neutrophils (IL-17α) and cDC (CXCL12: ligand for CXCR4; CCL3 & CCL5: ligand for CCR5; CCL2: ligand for CCR2) [41], [42]. Cultures from *Cxcr5−/−* mice produced a 2 to 10-fold increase in the level of most of cytokines and chemokines analyzed (Fig. 4B). We then examined supernatants depleted with α-GalCer-tetramer and blocked with anti-CD1d antibody (designated “treated” groups) to deplete all NKT cells. We found that the combination of depletion+blocking of CD1d function reduced cytokines and chemokine levels which were found to be at or below that of WT cultures (Fig. 4B). Only secretion of IL-6 was partially reduced by NKT cell depletion. Increased cytokine and chemokine levels we observed here could be produced directly by activated NKT cells or their interaction with other cell types, such as dendritic cells, as shown by others [43]. These data suggest that *C. muridarum* activates NKT cells which recruits early inflammatory cells, particularly neutrophils and activated cDC to the GT.

### *C. muridarum* activates iNKT and type II NKT cells and the lack of NKT cells in *Cd1d−/−* mice reduces UGT pathology

**Figure 5:**
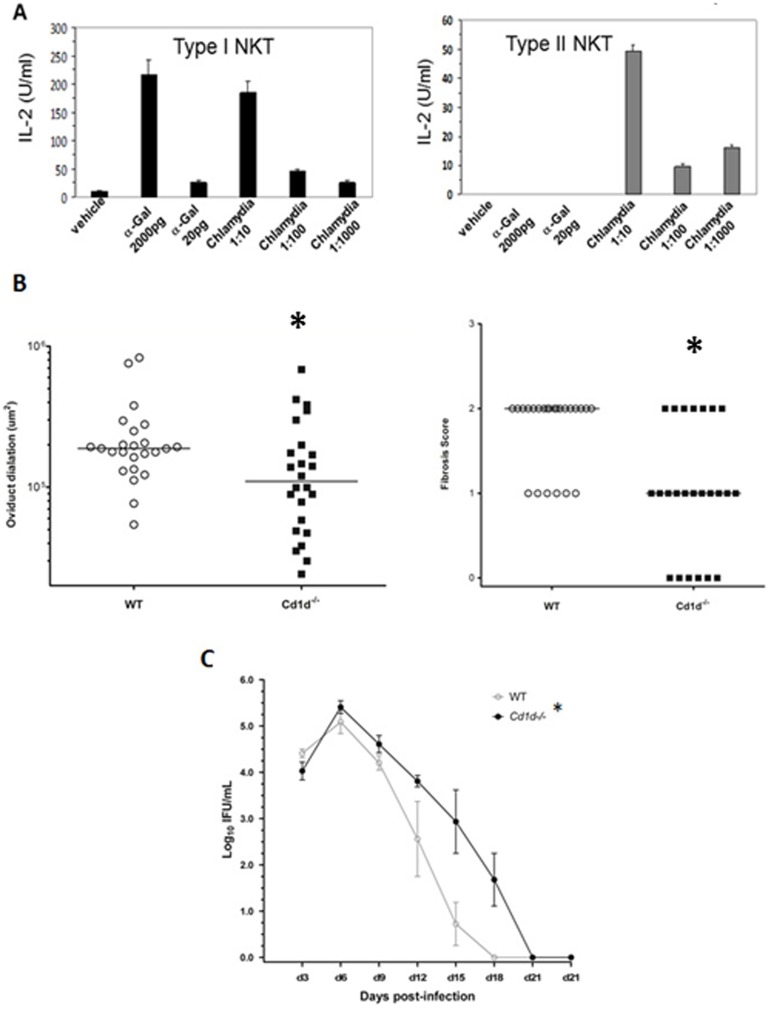
*C. muridarum* activates iNKT & type II NKT cells in vitro and in vivo. (A) Various dilutions of *C. muridarum* sonicate or α-Gal was added to cultures of type I NKT cell hybridoma 1.2 and a type II NKT cell hybridoma 1.9. IL-2 was measured in triplicate by ELISA. Experiments were and repeated 2–3 times. (B) Scatter plot of oviduct diameter and fibrosis scores from WT mice and *Cxcr5−/−* mice obtained from hematoxylin and eosin and trichrome stained slides, respectively. *p<0.05, Mann-Whitney, n = 6 mice or 12 oviducts/group. (C) Vaginal swabs were collected throughout the course of infection in WT and *Cd1d−/−* mice. *p<0.01 by 2-way repeated measures, n = 6 mice/group.

We determined if chlamydial organisms contained antigens which directly activated iNKT and II NKT cells in a cell-free system as described [32]. Our analysis found that a *C. muridarum* sonicate induced IL-2 secretion and the activation of murine NKT hybridomas. Figure 5A shows one of two iNKT cell hybridoma Vα14Vβ8.2 (1.2, 2C12) and a type II NKT cell hybridoma (19) [44]. The finding from this assay, which contains only a TCR ligand presented on CD1d molecules and is free of any antigen presenting cells, indicates that *C. muridarum* organisms contain glycolipid antigens which can bind and activate iNKT and II NKT cells. It is likely that *C. muridarum* contains at least 2 glycolipids since type II NKT cells do not recognize iNKT cell ligands [19]. The increased bacterial burden seen early after infection in *Cxcr5−/−* mice (Fig. 1B) could contribute to the activation of NKT cells. *Cd1d−/−* mice, which do not contain any NKT cells, were given a genital infection with *C. muridarum* to determine whether their absence influences oviduct dilation, fibrosis or bacterial burden in the GT. We found that oviduct dilation and fibrosis were significantly reduced in mice that lacked NKT cells (Fig. 5B). Monitoring bacterial burden with vaginal swabs showed that *Cd1d−/−* mice had an increase in bacterial burden (Fig. 5C) and confirms that NKT cells are involved in *C. muridarum* genital infection as previously shown using *Cd1d−/−* mice [45].

#### CXCR5 +10950 T>C (rs3922) correlates with lack of tubal infertility following C. trachomatis infection in humans

**Figure 6:**
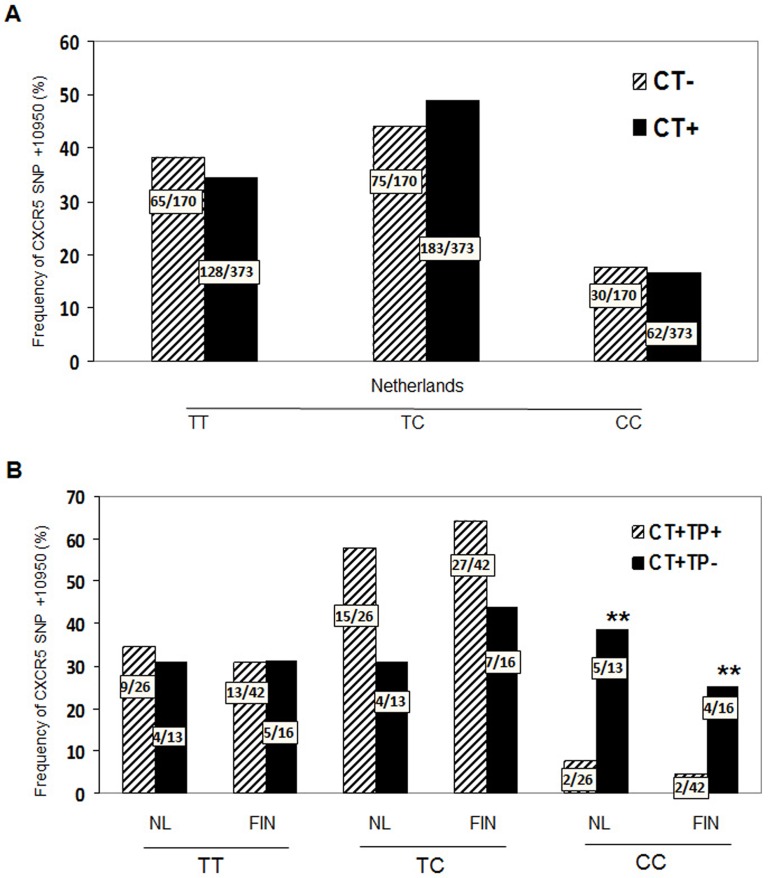
Distribution of the *CXCR5* SNP+10950 T>C (rs3922) in all three cohorts. Genomic DNA was extracted from peripheral blood. Distribution of *CXCR5* SNP+10950 T>C (rs3922) was determined in samples (A) women with and without a positive *C. trachomatis* PCR on cervical swab, n = 543 or (B) women from The Netherlands (n = 56, p: 0.03; OR 0.1, 95%CI: 0.02–0.82) and Finland (n = 114, *p*: 0.04; OR 0,2; 95%CI: 0.2–0.9) that were positive for *C. trachomatis* and clinical evidence of subfertility. Statistics for both groups combined (*p*: 0.002; OR = 0.1; 95%CI: 0.04–0.5.9) by χ2 and Fisher Exact test, (CT: *C. trachomatis;* TP: Tubal pathology; NL: Netherlands, FIN: Finland.

There is considerable variability in both susceptibility to acquiring a *C. trachomatis* infection and development of GT pathology following infection [36], [46], [47]. To determine if the genetic variation in the *CXCR5* gene contributes to this variability in *C. trachomatis* infected humans we undertook a single-polymorphic nucleotide (SNP) analysis for genetic variation in the *CXCR5* gene using TaqMan analysis. We examined *CXCR5* SNPs in 1940 females from The Netherlands and Finland with chlamydial genital infection. We analyzed three SNPs in the human *CXCR5* gene; +3439 C>T, (rs497916), +9086 T>C, (rs12363277) and +10950 T>C (rs3922) which tag 96.6% of the haplotypes spanning 7.6 KB (∼41%) of the *CXCR5* gene. The distribution of the SNPs and haplotypes in our study population is shown in Tables S1 and S2. We evaluated these SNPs for contribution to the susceptibility of *C. trachomatis* genital infection by comparing women attending a STD clinic for potential uncomplicated *C. trachomatis* infection. As shown in Figure 6A, carriage of *CXCR5* +10905 T>C (rs3922) did not differ significantly between women with and without *C. trachomatis* genital infection. However, other alleles did influence susceptibility since carriage of the *CXCR5* +9086 T>C (rs12363277) allele was significantly reduced in *C. trachomatis* infected women compared to controls (Table S1). *C. trachomatis* infected women had a significantly lower frequency of the *CXCR5* haplotype III and a trend of lower frequency in CXCR5 haplotype IV compared to CT negative women (Table S2). These results suggest that women carrying CXCR5 haplotype III to have reduced severity of *C. trachomatis* infections while the *CXCR5* +10950 (rs3922) SNP does not contribute to susceptibility of acquiring a genital infection with *C. trachomatis*.

We next evaluated whether different variants of the *CXCR5* gene correlated with the development of tubal pathology following chlamydial genital infection. We identified that *CXCR5* +10950 T>C (rs3922) was differentially distributed between women who developed tubal infertility following *C. trachomatis* infection versus those that did not following CT infection. Women developing tubal infertility had a statistically significant decrease in the frequency of *CXCR5* +10950 CC, both in the cohort from the Netherlands and the cohort from Finland (Fig. 6B). Possessing *CXCR5* SNP +10950 (rs3922) with base pair CC, appears to protect against development of tubal pathology following infection. Although the functional consequences, i.e., gain or loss of CXCR5 chemokine function with SNP +10950 T>C (rs3922) is not known, the data presented here are consistent with the murine data and suggest that the *CXCR5* gene influences chlamydial genital infection and tubal pathology in humans.

## Discussion

We have identified a novel form of immune regulation in the GT that depends on the chemokine receptor, CXCR5, in mice and humans. In the absence of this receptor, NKT cell activation is increased following *C. muridarum* infection of mice and results in increased cytokine and chemokine production which could attract increased numbers of neutrophils and activated cDC to the GT early after infection. Although *Cxcr5−/−* mice had increased chlamydial burden in the GT, equal numbers of EB were used to activate NKT cells *in vitro* in WT and *Cxcr5−/−* mice and there was still significant NKT cell activation as shown by IFNγ production. Neutrophils have been shown to induce GT inflammation and fibrosis and although we do not find occlusion fibrosis, we do find differences in oviduct dilation in the absence of NKT cells [6], [7]. The increased number of activated cDC could contribute to the increased numbers of T cells found in the oviducts and increased chronic inflammatory scores measured in the UGT.. Although CXCR5 is expressed on a subset of NKT cells (Fig. S1) [48], CXCR5 could be expressed by another cell type which impacts NKT cell activation and will be investigated in future studies.

Activation of NKT cells induces numerous functions including interaction with other cell types as shown in *Chlamydia* infections [49]. Our data in *Cxcr5−/−* mice shows an increase in iNKT cell activation as defined by expression of CD69. This difference is confined to activated iNKT cells since we did not find a difference in the total number of NKT cells between *Cxcr5−/−* and WT mice. Also, the lack of CXCR5 did not prevent trafficking of activated iNKT cells to the GT indicating that CXCR5 is not present on the subset of NKT cells in the GT or that other chemokine receptors compensate. In addition, our data imply that signaling thorough CXCR5 is important for regulating the activation state and cytokine/chemokine secretion of iNKT cells. There are precedents for a proposed role for a chemokine receptor in regulating iNKT cell activity. For example, CXCR6 and its ligand have been shown to regulate the homeostasis of iNKT cells in the liver and trafficking to that site was not affected [50].

The presence of CD1d in the GT of humans and the finding that it is a target of degradation by the chlamydiae CPAF suggests that NKT cells are important cells to regulate during genital infection [51], [52]. Bilenki, L. *et al.* [45] showed that activated iNKT cells enhanced chlamydial growth following infection of the mouse pathogen, *C. muridarum* which was reversed in *CD1d−/−* mice. They found that activation of iNKT cells induced a Th2-type cytokine milieu which enhanced *C. muridarum* lung infection, delayed eradication of the organism including development of a Th1 response and induced lung inflammation [45]. These findings support our work in *Cd1d−/−* mice. In contrast, our findings differed from a study by Wang et. al., which showed that activation of iNKT with α-GalCer during genital infection increased production of Th1 cells and slightly reduced bacterial burden but there was no data on UGT pathology or use of knockout mice [14]. This can be explained by comparing α-GalCer stimulation which is not as conclusive as studies in *Jα18−/−* mice (target on iNKT cells) and those in *Cd1d−/−* mice (target all NKT cell subsets) which target different subsets of NKT cells. Also, the many subsets of NKT cells may be functioning at the same time during *C. muridarum* infection which additionally confounds the information among various models. We also found direct evidence that *C. muridarum* infection activates iNKT cells and it was recently reported that *C. muridarum* glycolipid exoantigen (GLXA) also activated iNKT cells in a CD1d-cell free assay [13]. Future studies are planned to identify these antigens using mass spectrophotometric techniques.

CXCR5 is expressed on B cells, T helper follicular cells and NKT cells [53] which localize in germinal centers and are important for antibody class switching by conventional or B-2 B cells [54]. As expected *Cxcr5−/−* mice given a genital infection with *C. muridarum* produce significantly less *C. muridarum* specific IgG showing that these mice have defective T-dependent and/or T-independent antibody production (data not shown). We also compared the number of CD4 cells in spleens of WT and *Cxcr5−/−* to determine whether the lack of CXCR5 impacts the CD4 cell compartment. The percent of CD4 cells was slightly increased in *Cxcr5−/−* mice even before infection and more profoundly after infection suggesting that although the number of T helper follicular cells was absent in *Cxcr5−/−* mice this did not markedly reduce number of CD4 T cells in the T cell compartment before and after infection.

This is the first study to provide direct evidence that antigens contained within chlamydial EB directly activate type II NKT cells subsets. iNKT and type II NKT cells are activated by *C. muridarum* EB suggesting that they contain at least two glycolipid antigens since iNKT and type II NKT have diverse TCR [55]. After entering host cells, chlamydiae intercept the host exocytic pathway and acquire various types of host lipids (phosphatidylinositol, phosphatidylcholine, cardiolipin and sphingolipids) for incorporation into bacterial cell walls and inclusion membrane and are necessary for growth [56]–[58]. Chlamydiae also contain enzymes that modify host-derived single chain fatty acids to the microbial form of branched chain fatty acids [59]. Additional studies have shown that chlamydiae can synthesize limited phospholipids: phosphatidylethanolamine, phosphatidylserine and phosphatidylglycerol including a CDP-diacylglycerol [60]. It will be important to identify chlamydial glycolipids that are presented by CD1d to define how they influence genital infection.

We have also shown a similar influence of CXCR5 on tubal pathology in humans. Women carrying the variant allele of *CXCR5* +10950 CC did not develop the level of tubal pathology seen in subfertile women after CT infection. Interestingly, humans with the *CXCR5* SNP+10950 T>C (rs3922), were equally susceptible to *C. trachomatis* genital infection compared to carriers of *CCR5* SNPs [34]. Other studies have also found a SNP analysis of other chemokine genes correlates to tubal pathology. Barr *et. al.*, found that the CCR5-related inflammatory response was demonstrated to be crucial for the development of tubal factor infertility [34]. They showed that in women with anti-chlamydial IgG responses, tubal pathology correlated with a low incidence of functional *CCR5*Δ32 deletion (7%), while women without tubal pathology had higher incidence of the *CCR5*Δ32 deletion (31%). Thus, in mice and humans the inflammation associated with CXCR5 and CCR5 function may predispose to development of complications of chlamydial infection, such as tubal factor infertility.

The host response is determined by interaction of pathogen-associated molecular patterns (PAMP) of chlamydiae and components of the innate host response. This interaction in turn, is based on individual genetic variation. Other studies have identified additional genes which also contribute to the variability in acquiring a chlamydial infection and immunopathology. Recently, Bailey *et. al.* [61] estimated the relative contribution of host genetics to the total variation in lymphoproliferative responses to chlamydial antigens by analysing these responses in 64 Gambian pairs of twins from trachoma-endemic areas. Proliferative responses to serovar A EB antigens were estimated in monozygotic and dizygotic twin pairs. They found a stronger correlation and lower within-pair variability in these responses in monozygotic compared to dizygotic twin pairs. The heritability estimate was 0.39, suggesting that host genetic factors contributed almost 40% of the variation. This study reinforces the concept of compiling genetic traits that influence infection and genital tract pathology for the development of a sub-fertility risk profile to advance disease prevention profiles and identify individuals for immune-modulatory therapeutics. This approach is currently the focus of EpiGenChlamydia in Europe.

There is a strong association between repeated *C. trachomatis* genital infection and development of tubal inflammation and infertility [4], [5]. A combination of bacterial factors, host factors, epidemiologic and demographic factors, such as co-infections, and the mutual interaction of these factors influence this association [62]. Additionally, there are reported inter-individual differences in the clinical course of *C. trachomatis* genital infection such as; transmission, symptoms, persistence or clearance of infection, and the development of late complications [4], [63], [64]. It is difficult to correlate these to persistence of infection, especially when the detected serovar is not identical to the previous one [63]–[66]. Possibly repeated activated of NKT cells with multiple serovars following chlamydial genital infection in those with altered in CXCR5 expression and function could be responsible for inter-individual differences. There is great variability in the frequency of Vα24-Jα18 iNKT cells in the peripheral blood of humans (0.001–3.0%) and this suggests that differences occur in iNKT cell development, maturation and/or differentiation [67], [68]. It is possible that frequency and type of infections plus genetic differences in *Cxcr5* gene influence iNKT cell activation in humans [12]. This knowledge will be beneficial for the design of therapeutic strategies and vaccine development against *Chlamydia* infection.

Our study is the first describing the protective effect of *CXCR5* gene polymorphisms in the development of *C. trachomatis* infection and late complications of this disease. In this genetically exploratory study using well defined clinical cohorts and only one gene, having an *in vitro* proof of association, we had decided not to “overdo” by using the most stringent statistical approach. The additional strength on this study is that our genetic association is confirmed in a second independent cohort, something which is in most studies is not even done. As we have shown in the knockout mouse model, an increased NKT activity leads to an increased bacterial burden in the GT and late complications of this disease. In humans, carriage of the variant allele, +10950 T>C, might result in an altered function of the *CXCR5* gene, and thereby an altered expression of NKT cells thus leading to fewer late complications of *C. trachomatis* infections such as tubal pathology. It is not known if +10950 T>C SNP of *CXCR5* gene results in a gain or loss of function. Although the exact pathways and mechanism(s) need to be elucidated, *Cxcr5* knockout mice appear as a viable model for understanding the pivotal role of CXCR5. We therefore hypothesize that in humans, the mechanisms underlying the course of disease are similar as in *Cxcr5* knockout mice. Therefore, more studies are needed of human *CXCR5* genetic polymorphisms on the function of this chemokine receptor.

## Supporting Information

Table S1 **Distribution of the CXCR5 SNPs in an STD cohort from Amsterdam two tubal pathology cohorts.** Genomic DNA was extracted from peripheral blood and PCR was performed for three CXCR5 SNPs; +3439 C>T (rs497916), +9086 T>C (rs12363277), and +10950 T>C (rs3922). ap:0.0487; OR 0.5, 95%CI: 0.3–1,0; bp: 0.03; OR 0.1, 95%CI: 0.02–0.82; cp: 0.04; OR 0,2; 95%CI: 0.2–0.9. Groups were compared using χ2 and Fisher Exact test, where appropriate. p<0.05 was considered statistically significant. CT: C. trachomatis; TP: Tubal pathology.
(PDF)

Table S2 **Haplotypes of CXCR5 in in two tubal pathology cohorts and an STD cohort from Amsterdam.** Genomic DNA was extracted from peripheral blood and PCR was performed for three CXCR5 SNPs; +3439 C>T (rs497916), +9086 T>C (rs12363277) and +10950 T>C (rs3922). CXCR5 haplotypes were inferred using PHASE v2.1.1 [62], [63] and SNPHAP [64]. ap:0.0097, OR: 0.18, 95% CI: 0.05–0.66. Groups were compared using χ2 and Fisher Exact test, where appropriate. p<0.05 was considered statistically significant. Haplotype IV was not significantly different from CT- from the FCT: C. trachomatis; TP: Tubal pathology.
(PDF)

Figure S1 **7.5% of NKT cells express CXCR5.** Representative dotplots showing NKT cell gating and expression of CXCR5, 7 days after infection. Single cell suspensions of lymphocytes were stimulated with PMA and ionomycin, and stained for NKT cells using α-GalCer tetramer, CD3, CD4, CD8, NK1.1, CD69 and CXCR5. Dotplots were gated on CD3+, NK1.1+, -GalCer-tetramer+ cells.
(TIF)
