# Correction: Interruption of CXCL13-CXCR5 Axis Increases Upper Genital Tract Pathology and Activation of NKT Cells following Chlamydial Genital Infection

**DOI:** 10.1371/annotation/2b6aab70-3c92-490e-86f0-ae15787dfa6e

**Published:** 2013-05-24

**Authors:** Janina Jiang, Ouafae Karimi, Sander Ouburg, Cheryl I. Champion, Archana Khurana, Guangchao Liu, Amanda Freed, Jolein Pleijster, Nora Rozengurt, Jolande A. Land, Helja-Marja Surcel, Aila' Tiitinen, Jorma Paavonen, Mitchell Kronenberg, Servaas A. Morré, Kathleen A. Kelly

There was an error in the Funding statement.

The correct version of the statement is:

The work was sponsored by grants from the National Institutes of Health, AI026238 (KAK) and AI45053 (MK); Iris Cantor-UCLA Women's Health Center Executive Advisory Board, NCATS UCLA CTSI UL1TR000124 (KAK), plus a sponsored research award from Abraxis Bioscience, Inc (KAK). The human studies were performed by members of the EpiGenChlamydia Consortium funded by the European Commission within the Sixth Framework Program through contract no. LSHG-CT-2007-037637. The funders had no role in study design, data collection and analysis, decision to publish, or preparation of the manuscript. 

