# Supplementary material for: Interruption of CXCL13-CXCR5 Axis Increases Upper Genital Tract Pathology and Activation of NKT Cells following Chlamydial Genital Infection
Source: PLoS One. 2012 Nov 26;7(11):e47487. doi: 10.1371/journal.pone.0047487 (PMC3506621; doi:10.1371/journal.pone.0047487)
Supplement: Table S1 — Distribution of the CXCR5 SNPs in an STD cohort from Amsterdam two tubal pathology cohorts. Genomic DNA was extracted from peripheral blood and PCR was performed for three CXCR5 SNPs; +3439 C>T (rs497916), +9086 T>C (rs12363277), and +10950 T>C (rs3922). ap:0.0487; OR 0.5, 95%CI: 0.3–1,0; bp: 0.03; OR 0.1, 95%CI: 0.02–0.82; cp: 0.04; OR 0,2; 95%CI: 0.2–0.9. Groups were compared using χ2 and Fisher Exact test, where appropriate. p<0.05 was considered statistically significant. CT: C. trachomatis; TP: Tubal pathology. (PDF) [file pone.0047487.s001.pdf]

		+3439 C>T						+9086 T>C						+10950 T>C					
	n	CC	%	CT	%	TT	%	TT	%	TC	%	CC	%	TT	%	TC	%	CC	%
STD cohort																			
CT+	170	84	49.4	69	40.6	17	10.0	159	93.5	11	6.5	0	0.0[a]	65	38.2	75	44.1	30	17.7
CT-	373	185	49.6	158	42.4	30	8.0	328	87.9	44	11.8	1	0.3	128	34.3	183	49.1	62	16.6
Tubal Pathology																			
The Netherland																			
CT+TP+	26	16	61.5	9	34.6	1	3.9	23	88.5	3	11.5	0	0.0	9	34.6	15	57.7	2	7.7[b]
CT+TP-	13	7	53.9	4	30.8	2	15.4	13	100.0	0	0.0	0	0.0	4	30.8	4	30.8	5	38.5
Finland																			
CT+TP+	42	16	38.1	25	59.5	1	2.4	40	95.2	2	4.8	0	0.0	13	31.0	27	64.3	2	4.7[c]
CT+TP-	16	8	50.0	5	31.3	3	18.8	15	93.8	1	6.3	0	0.0	5	31.3	7	43.8	4	25.0

Table S1. Distribution of the *CXCR5* SNPs in an STD cohort from Amsterdam two tubal pathology cohorts. Genomic DNA was extracted from peripheral blood and PCR was performed for three *CXCR5* SNPs; +3439 C>T (rs497916), +9086 T>C (rs12363277), and +10950 T>C (rs3922). [a]P:0,04; OR 0,4; 95%CI: 0,5-1,0; [b]P: 0,03; OR 0,1 95%CI: 0,02-0,85; [c]P: 0,04; OR 0,2; 95%CI: 0,2-0,9. Groups were compared using $\chi 2$ and Fisher Exact test, where appropriate. $p<0.05$ was considered statistically significant. CT: *C. trachomatis;* TP: Tubal pathology.
