# Supplementary material for: Interruption of CXCL13-CXCR5 Axis Increases Upper Genital Tract Pathology and Activation of NKT Cells following Chlamydial Genital Infection
Source: PLoS One. 2012 Nov 26;7(11):e47487. doi: 10.1371/journal.pone.0047487 (PMC3506621; doi:10.1371/journal.pone.0047487)
Supplement: Table S2 — Haplotypes of CXCR5 in in two tubal pathology cohorts and an STD cohort from Amsterdam. Genomic DNA was extracted from peripheral blood and PCR was performed for three CXCR5 SNPs; +3439 C>T (rs497916), +9086 T>C (rs12363277) and +10950 T>C (rs3922). CXCR5 haplotypes were inferred using PHASE v2.1.1 [62], [63] and SNPHAP [64]. ap:0.0097, OR: 0.18, 95% CI: 0.05–0.66. Groups were compared using χ2 and Fisher Exact test, where appropriate. p<0.05 was considered statistically significant. Haplotype IV was not significantly different from CT- from the FCT: C. trachomatis; TP: Tubal pathology. (PDF) [file pone.0047487.s002.pdf]

CXCR5 Haplotypes											
	2n	I CTT	%	II TTC	%	III CTC	%	IV TCC	%	V TTT	%
STD cohort											
CT+	340	204	60.0	91	26.8	33	9.7	11	3.2[a]	1	0.3
CT-	746	438	58.7	171	22.9	90	12.1	46	6.2	1	0.1
Tubal Pathology											
The Netherland											
CT+TP+	52	32	61.5	7	13.5	9	17.3	3	5.8	1	1.9
CT+TP-	26	12	46.2	8	30.8	6	23.1	0	0.0	0	0.0
Finland											
CT+TP+	84	53	63.1	25	29.8	4	4.8	2	2.4	0	0.0
CT+TP-	32	17	53.1	7	21.9	7	21.9	1	3.1	0	0.0

Table S2. Haplotypes of *CXCR5* in in two tubal pathology cohorts and an STD cohort from Amsterdam. Genomic DNA was extracted from peripheral blood and PCR was performed for three *CXCR5* SNPs; +3439 C>T (rs497916), +9086 T>C (rs12363277) and +10950 T>C (rs3922). *CXCR5* haplotypes were inferred using PHASE v2.1.1 [62,63] and SNPHAP [64]. [a]P:0.04; OR: 0.4; 95%CI:0.2–1.0, groups were compared using $\chi 2$ and Fisher Exact test, where appropriate. $p<0.05$ was considered statistically significant. CT: *C. trachomatis;* TP: Tubal pathology.
