# Supplementary figures and images for: Interruption of CXCL13-CXCR5 Axis Increases Upper Genital Tract Pathology and Activation of NKT Cells following Chlamydial Genital Infection
Source: PLoS One. 2012 Nov 26;7(11):e47487. doi: 10.1371/journal.pone.0047487 (PMC3506621; doi:10.1371/journal.pone.0047487)

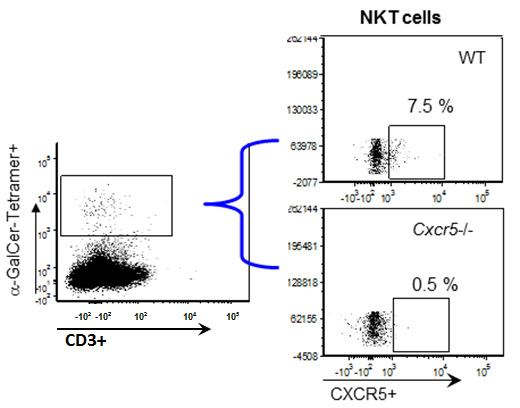

Supplement: Figure S1 — 7.5% of NKT cells express CXCR5. Representative dotplots showing NKT cell gating and expression of CXCR5, 7 days after infection. Single cell suspensions of lymphocytes were stimulated with PMA and ionomycin, and stained for NKT cells using α-GalCer tetramer, CD3, CD4, CD8, NK1.1, CD69 and CXCR5. Dotplots were gated on CD3+, NK1.1+, -GalCer-tetramer+ cells. (TIF) [file pone.0047487.s003.tif]
